# Lobular Breast Carcinoma Mimicking a Primary Gastric Malignancy

**DOI:** 10.7759/cureus.40371

**Published:** 2023-06-13

**Authors:** Ricardo Anguiano-Albarran, Franklin Obi, Sidart Pradeep, Daniel Cain, Bryan Bartlett, Melvin Simien

**Affiliations:** 1 Internal Medicine, Baylor Scott & White All Saints Medical Center, Fort Worth, USA; 2 Pathology, Baylor Scott & White All Saints Medical Center, Fort Worth, USA; 3 Gastroenterology and Hepatology, Baylor Scott & White All Saints Medical Center, Fort Worth, USA; 4 Interventional Endoscopy, Baylor Scott & White Digestive Diseases – Fort Worth, Fort Worth, USA

**Keywords:** endoscopy, gastric cancer, metastatic, breast cancer, lobular carcinoma

## Abstract

Invasive lobular carcinoma (ILC) is the second most common subclass of breast cancer and adds to the breast malignancy burden in women.^ ^Studies focused on metastatic patterns of ILC have reported bone, gynecologic organs, the peritoneum, and the gastrointestinal tract as potential sites of metastasis. Metastatic spread to the stomach has been reported, but generally remains an infrequent finding. Due to vague symptomatology and the visual limitations of endoscopic examination, metastatic lesions can often mimic a primary gastric malignancy. Metastasis in the stomach can be challenging to diagnose and requires a multimodal, thorough endoscopic and immunohistochemical evaluation. It is important to distinguish the primary origin of malignant lesions as treatment can range from systemic chemotherapy to surgical resection based on the diagnosis. We present a case of an underlying ILC metastatic lesion mimicking a primary gastric adenocarcinoma.

## Introduction

Up until the late 1980s, stomach cancer was the second most frequent cause of cancer-related deaths worldwide [1]. Since then, rates of gastric cancer have dropped due to advances in screening, treatment, and preventative education [1,2]. Despite these advancements, stomach cancer mortality remains unchanged and is among the top five diagnosed malignancies. In both sexes, stomach cancer closely follows lung, liver, and colorectal cancer in overall mortality [3,4]. In females, stomach cancer conveys an approximate 6.5% mortality vs 15% mortality for breast cancer. In males, stomach cancer mortality is about 9.5% [4].

Invasive lobular carcinoma (ILC) is the second most common subclass of breast cancer and accounts for roughly 5% to 15% of the breast malignancy burden in women [5]. In women with a history of invasive ILC before age 50, genetic testing for abnormal changes in the CDH1 gene should be considered. Inactivating mutations in the CDH1 gene have previously been identified in association with an increased risk of hereditary diffuse gastric cancer and ILC [6]. Studies focused on metastatic patterns of ILC have reported bone, gynecologic organs, the peritoneum, and the gastrointestinal tract as potential sites of metastasis [5,7]. Metastasis in the stomach is difficult to detect since it can present very similarly to primary gastric cancer. Endoscopy and immunohistology are essential for characterizing the tumor so that the correct therapy can be initiated. The following is a case of ILC that mimicked a primary gastric adenocarcinoma. 

## Case presentation

**Figure 1:**
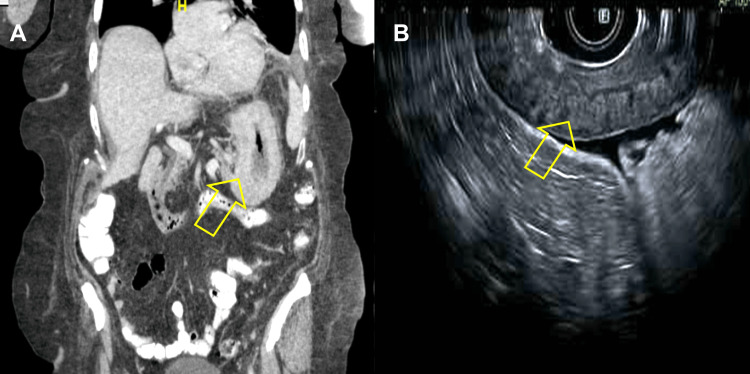
Coronal CT (A) and EUS (B) view demonstrating extensive stomach wall thickening EUS: Endoscopic ultrasound

**Figure 2:**
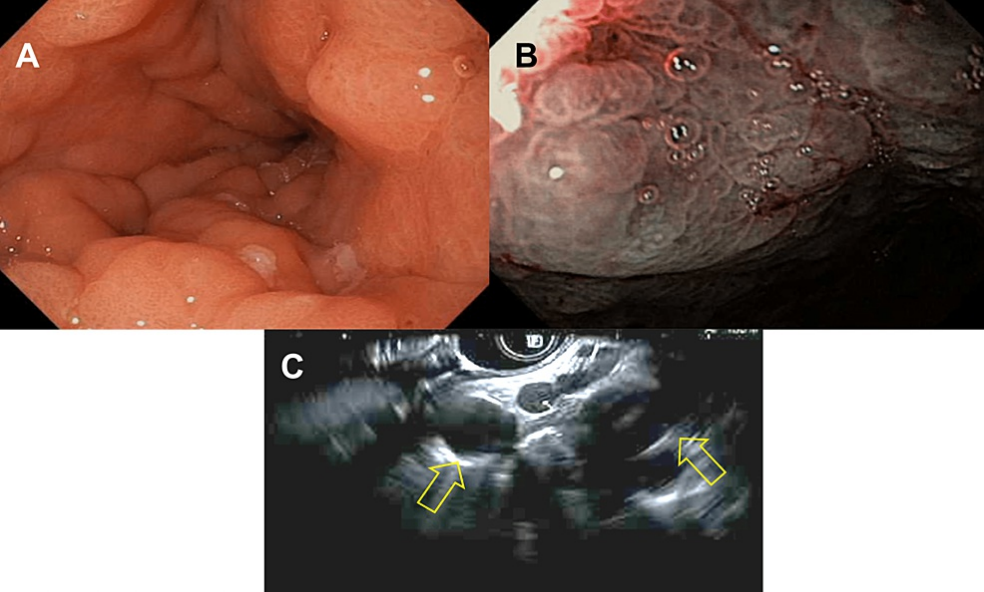
Endoscopic gross images and EUS A: Endoscopic longitudinal view of the antrum; B: Narrow-band imaging view of the distorted gastric mucosa; C: The EUS view identifying multiple lymph nodes EUS: Endoscopic ultrasound

Our patient was a 65-year-old female with a medical history of myotonic dystrophy, deep vein thrombosis on chronic anticoagulation, ulcerative colitis, and breast lobular carcinoma two years status post left mastectomy and radiation therapy. Her previous breast cancer was estrogen receptor (ER) positive, progesterone receptor (PR) negative, and human epidermal growth factor receptor 2 (HER2) negative. Family history was significant for a mother who had breast cancer. She presented with complaints of heart palpitations, fatigue, and an unintentional 30-pound weight loss over four months. In addition, she had been experiencing severe reflux limiting oral intake for the past four months. Computed tomography imaging of the abdomen and pelvis demonstrated new trace volume pelvic ascites with peritoneal nodularity, new omental fat stranding suggestive of edema and caking, new osseous sclerotic lesions concerning for metastatic disease, and thickening of the stomach wall (Figure 1A). Due to elevated tumor marker levels, CT findings, and weight loss, the patient underwent esophagogastroduodenoscopy (EGD) with endoscopic ultrasound (EUS). The EGD revealed Los Angeles (LA) grade C erosive esophagitis and inflamed gastric mucosa. Mucosal biopsies were taken and sent to pathology. The EUS identified several enlarged lymph nodes in the aortopulmonary region, and stomach wall thickness was measured at approximately 20 mm (Figure 1B, and Figure 2).

Given the stomach findings and the patient’s persistent gastric reflux in the setting of the weight loss, she was referred to gastroenterology. By the time she met with the gastroenterologist, she had lost an additional 15 pounds and was having nausea with vomiting after eating solid food. She was started on an oral proton pump inhibitor, but no relief of symptoms occurred. Additional labs were also ordered which revealed significant lab results and tumor markers (Table 1). The primary concern was now the elevated tumor markers in addition to liver enzymes. 

**Table 1:** Liver enzyme and tumor marker values

Lab	Patient value	Reference range
Aspartate aminotransferase (AST)	525 IU/L	0-40 IU/L
Alanine aminotransferase (ALT)	583 IU/L	0-32 IU/L
Alkaline phosphatase (ALP)	201 U/L	44-121 IU/L
Cancer antigen 19-9	78 U/mL	<34 U/mL
Cancer antigen 125	163 U/mL	1-35 U/mL
Carcinoembryonic antigen	30.8 ng/mL	0-2.4 ng/mL

Initial microscopic analysis of the biopsy specimens demonstrated a tumor composed of small-to-intermediate sized polygonal cells with variable oval nuclei with irregular finely stippled chromatin and variable nucleoli. Some examined cells exhibited prominent mucinous droplets in their cytoplasm that resulted in the peripheral displacement of their nucleoli. the HER2 stain screening was negative, which prompted concern for a primary gastric malignancy. Based on these findings, an initial diagnosis of poorly cohesive signet ring cell carcinoma was made.

**Figure 3:**
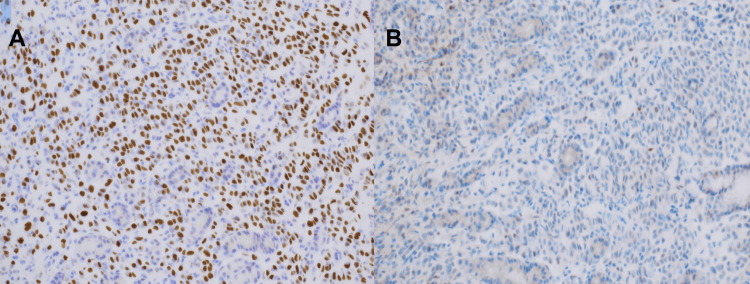
Histological staining of gastric tumor A: The GATA3 stain demonstrates uniform positivity, indicating primary lobular carcinoma; B: The CDX2 stain is uniformly negative excluding primary stomach adenocarcinoma

However, due to the patient’s prior history of breast cancer, further immunohistochemical analysis with special stains was requested. The CK7 staining was found to be strongly positive, the GATA3 stain was positive in a nuclear fashion, and the CDX2 staining was negative (Figure 3). These staining patterns, instead, indicated a primary source of lobular breast carcinoma. Samples were ER-positive, and PR and HER2 negative. Due to the patient’s advanced metastatic disease and overall declining clinical status, the patient opted to transition to hospice care.

## Discussion

In this patient with a previous cancer history, determining the etiology of new lesions was important. The amendment to the final diagnosis highlights the challenges of immunohistochemical analysis of malignant lesion specimens. Initial analysis revealed mucin-containing cytoplasm resulting in displacement and distortion of the nucleus, a presentation classically consistent with signet ring cell carcinoma. The HER2 stain per protocol was negative as well. Thus, the initial evaluation was consistent with a mucinous, primary gastrointestinal malignancy.

The CK7, GATA3, and CDX2 stains suggested ILC. The GATA3 is involved in the luminal differentiation of breast epithelium and can be utilized to differentiate primary breast tumors from primary gastric malignancy. The CDX2 is a nuclear transcription factor critical for intestinal embryonic development and is specific for the identification of malignancy of gastrointestinal origin [8,9].

This case demonstrates the importance of thorough immunohistochemical analysis in diagnosing metastatic stomach lesions. The appearance of ILC on histology can be variable depending on the subtype such as classic type, pleomorphic, histiocytoid, signet ring, and tubulolobular [10]. Workup aims to identify GATA3 and CK7 expression as they are closely associated markers of mammary origin [8,11]. Unfortunately, the CK7 imaging sample was unretrievable and we were unable to capture this for the purposes of this article.

## Conclusions

Though this case had a poor prognosis and limited treatment options at the time of diagnosis, it is a valuable addition to the literature as it showcases the importance of having breast metastasis included in the differential of gastric neoplasm workups. The significance of immunohistochemical analysis and the utility of EUS in this workup should also be viewed and emphasized when evaluating these tumors. 
